# The Academic Threat Appraisal Ratio Scale (ATARS): Insights into attainment, academic progression, and retention in higher education

**DOI:** 10.1111/bjep.12780

**Published:** 2025-05-03

**Authors:** Simon Cassidy

**Affiliations:** ^1^ University of Salford Salford UK

**Keywords:** academic progression, attainment, challenge‐threat, higher education, retention, threat appraisal

## Abstract

**Background:**

Previous studies examining threat appraisal and the influence of stress on human performance conclude that a *challenge* state leads to better performance than a *threat* state. Despite its potential, threat appraisal, particularly using self‐report measures, has been the subject of limited investigation in applied higher educational contexts.

**Aims:**

The study explored the potential of self‐report academic threat appraisal to explain academic progression and drop out in first‐year students and investigated associations between self‐report academic threat appraisal and relevant non‐cognitive factors.

**Sample:**

The sample comprised 186 first‐year undergraduate university students.

**Method:**

Students completed a newly adapted self‐report threat appraisal measure, the Academic Threat Appraisal Ratio Scale (ATARS), at the beginning of their degree course. End‐of‐year grade point average and academic progression were also measured along with self‐report measures of academic self‐efficacy, academic resilience, grit, and mindset.

**Results:**

Findings revealed that a significantly greater proportion of students eliciting a challenge state progressed at first attempt, and of those students failing to progress at first attempt, a significantly greater proportion had elicited a threat state (*χ* ^2^ (1) = 4.445, *p* = .035). Furthermore, academic threat appraisal was identified as a significant predictor of academic progression, while academic self‐efficacy was identified as a significant predictor of academic threat appraisal.

**Conclusions:**

Evidence supports self‐report academic threat appraisal as a significant factor in student attainment and academic progression in higher education, suggesting that the ATARS offers a relatively simple, valid, and scalable tool for early screening of students, enabling targeted student support.

## INTRODUCTION

Persistent high student drop out (non‐continuation) rates (Jack, 2023), particularly evident in the first year of university (van Rooij et al., 2017), and a continued focus on access, retention, attainment, and academic progression as key performance metrics in higher education (Hillman, 2024), make the search for explanatory factors and meaningful interventions a priority for the sector (Webb et al., 2017). This may, in part, explain evidence of intensified interest in factors other than university entry grades—determined by standardized tests such as A‐levels and SATs reflecting cognitive skills and ability—to help explain student achievement and success. Furthermore, in their review of the literature, Webb et al. (2017) note that ‘the likelihood of a student remaining in university can be attributed to a number of factors beyond their academic ability when they enter their first year’ (p. 5). Despite this, cognitive skills and ability involving reasoning and problem‐solving have traditionally provided the focus for work examining educative processes and student employment potential. However, a shift in more recent research towards recognizing the critical role played by non‐cognitive factors in educational achievement and success has been noted, together with the suggestion that their influence may be more important than that of cognitive factors (Khine, 2016). Whilst not devoid of cognitive processing (Borghans et al., 2008; Sommerfeld, 2011), non‐cognitive factors can be thought of as distinct from or less reliant on cognitive skills and abilities (i.e., non‐intellective, Magnano et al., 2020) and include a range of psychological constructs. The present study examines the significance of threat appraisal, involving perceived demands and resources, as one factor with the potential to contribute to a greater understanding of student attainment and academic progression in higher education.

### Non‐cognitive factors

A potential catchall for any factor other than standardized test scores, the term non‐cognitive has been applied broadly in the literature (Reynolds et al., 2021). However, prevalent examples of non‐cognitive skills and factors commonly encountered in the literature examining student success, and whose influence is well‐established, include self‐efficacy, grit, mindset, and more recently, academic resilience.

Self‐efficacy involves self‐judgements about perceived capabilities to execute a course of action required to achieve desired levels of performance and attain goals, and it is therefore involved in the regulation of perceived task difficulty, persistence, and effort (Honicke et al., 2023). Honicke et al. (2023) refers to ‘decades of research [that] highlight the positive effects of self‐efficacy on academic achievement’ (p. 1936). Examples includes McIlroy (2015) who report an association between academic self‐efficacy and academic performance in sixth form students, and one recent study and mediation analysis by Meng and Zang (2023) who report both direct and indirect (via academic engagement) effects of self‐efficacy on academic achievement in Chinese university students.

Conceptualized by Duckworth et al. (2007, p. 1087), grit is defined as ‘perseverance and passion for long‐term goals’ and is comprised of consistency of interests and perseverance of effort. It is unrelated to IQ, but essential to high achievement. Individuals high in grit are described as ‘gritty’, whose advantage [over those of equal intelligence] is stamina (Duckworth et al., 2007). Using path analysis, Alhabadi and Karpinski (2020) demonstrated a positive association (mediated by students' self‐efficacy) between grit dimensions ‘perseverance of effort ‘and ‘consistency of interest’ and self‐reported GPA in American university students. Grit has also been reported as an independent predictor of overall academic success in samples of Australian university health professional students (Carlo et al., 2022) and of student retention in samples of US military cadets (Duckworth et al., 2007). A recent review and meta‐analysis focusing on research conducted between 2007 and 2020 offers further evidence for the positive association between overall grit and academic achievement (Lam & Zhou, 2022).

The mindset concept relates to implicit theories of intelligence held by individuals. That is, intelligence viewed as fixed, unchanging and stable—a fixed or entity mindset—versus intelligence viewed as malleable, with the potential to develop and change—a growth or incremental mindset (Dweck, 1999). In their review and meta‐analysis of the influence of mindset on academic achievement, Costa and Faria (2018) conclude that the evidence supports a direct significant positive association between incremental (growth) mindset and academic performance, and provide an account of the characteristics of students who adopt a fixed mindset that helps illustrate the potential implications for education. This includes the tendency for fixed mindset students to adopt performance goals over learning goals, to prioritize assessment over learning to demonstrate ability and seek positive evaluations, to be less likely to select tasks perceived to be challenging, and to attribute poor performance to lack of ability so fail to increase effort or adopt effective remedial strategies when faced with challenges and setbacks. De Castella and Byrne (2015) provide evidence for the adverse effects of a fixed mindset in students, reporting a significant negative association between entity (fixed) mindset and self‐reported academic performance.

Finally, resilience, commonly defined by the capacity to ‘bounce back’, involves facing adversity with recovery to normal pre‐adversity levels of functioning (or even achieving growth) post‐adversity. Applied in an education context using context‐specific measures of resilience, academic resilience is defined as the ‘capacity to overcome acute and/or chronic adversity that is seen as a major threat to a student's educational development’ (Martin, 2013, p. 488) and is comprised of three contributing factors: increased perseverance; increased reflecting and adaptive help‐seeking; and increased avoidance of negative affect and emotional response (Cassidy, 2016). Exploring the association between resilience and student success in a 4‐year longitudinal study in a large sample of German students, Bittmann (2021) reports significant positive correlations between resilience and student self‐reported grades, and significant negative correlations between resilience and students' intention to drop out. These effects, demonstrating the association between resilience and better outcomes for students, were consistent at different points in the longitudinal study.

The evidence demonstrating an association between non‐cognitive factors and student achievement and success is convincing, with Frantz et al. (2022) referring to the growing body of literature specifically examining the pertinence of non‐cognitive factors and skills in relation to student retention. However, applying this knowledge and understanding in higher education practice, involving measuring and interpreting the gamut of potentially relevant non‐cognitive factors and skills in large student populations, is burdensome for both students and staff and may not be readily scalable in a meaningful or viable way at an institutional level. In short, pursuing whole‐university student retention interventions that are reliant on the assessment of non‐cognitive factors and skills measured using traditional psychometric tools is likely to prove a ‘hard sell’ to staff, students, and senior leaders as key stakeholders. There is merit, therefore, in exploring alternative, more pragmatic approaches that are feasible in the reality of everyday higher education practice yet still offer valid insight into factors related to student achievement and success. Threat appraisal, involving evaluation of perceived task demands against perceived personal resources and shown to predict future performance in other fields, may be one such alternative with the potential to offer greater utility in applied higher education practice.

### Threat appraisal

Underpinned by transactional models of stress such as that proposed by Lazarus and Folkman (1984), threat appraisal is an individual's subjective interpretation of a difficult or stressful situation according to perceived demands of the situation (*primary* appraisal) and perceived available personal resources to meet those demands (*secondary* appraisal). This leads to challenge and threat evaluations and associated challenge and threat states that influence an individual's motivation to action and performance (Krok et al., 2023; Vine et al., 2013, 2015). Threat appraisals are based on expectations regarding prospective, potentially stressful situations and events, so are, as Tomaka et al. (1993) note, a priori. Within a biopsychosocial model of challenge‐threat, Blascovich (2008) uses motivated performance situations, described as goal‐relevant and task engaging such as exams, to contextualize challenge‐threat as approach‐avoidance motivation, where ‘challenge results when an individual's evaluated resources outweigh situational demands and threat results when evaluated situational demands outweigh an individual's resources’ (p. 432). A threat state has been associated with higher self‐reported stress, differences in physiological reactivity between challenge and threat groups, and, critically, poorer task performance (Tomaka et al., 1993).

In a series of validation studies, Blascovich (2008) demonstrated the association between subjective and objective measures of threat appraisal. Both pre‐task self‐report evaluations of situational demands and personal resources (i.e., subjective measure of threat appraisal) and cardiovascular responses captured during task performance (i.e., objective measure of threat appraisal) were aligned with previously defined cardiovascular patterns of physiological toughness and weakness reported by Dienstbier (1989). While challenge‐threat studies in the main continue to rely primarily on physiological measures of threat appraisal (e.g., Smith et al., 2022), in a novel application of challenge‐threat, Vine et al. (2015) used a self‐report measure of threat appraisal to establish the relationship between stress response and performance in commercial airline pilots. Findings showed that in a highly stressful engine failure at take‐off simulation, pilots' performance (based on flight instructor assessment and simulator metrics) was associated with self‐report threat appraisal, where poorer performance was associated with self‐reported threat state. Similar results have been reported in studies using self‐report threat appraisal in other fields including competitive sport (Moore et al., 2013) and medical training (Vine et al., 2013), which Vine et al. cite, referring to consistent evidence that, when compared with a threat state, a challenge state predicts superior performance.

Whilst there are examples of the application of threat appraisal in academic and educational contexts, these are relatively limited, often restricted to the use of cardiovascular indices of threat appraisal, and with inconsistent or mixed findings. Seery et al. (2010), for instance, reported a significant association between cardiovascular markers that differentiate challenge and threat states and academic performance in undergraduate students, where relative challenge state predicted better end of term self‐reported GPA. Smith et al. (2022) on the other hand, also using only cardiovascular indices of challenge‐threat, failed to find a significant association between threat appraisal and academic performance using various measures of performance, including verbal and non‐verbal presentation skills and averaged annual module scores. Malkoc et al. (2023) offer one recent example where self‐report threat appraisal measures were employed. Using two adapted four‐item self‐report scales as separate measures of challenge and threat, Malkoc et al. reported an indirect relationship between threat appraisal and academic performance in Austrian university students during the COVID‐19 pandemic, where a threat state was positively related to unpleasant emotions, which in turn were directly negatively related to exam performance. Though focussing on fear appeals in high school maths exam performance, Putwain and colleagues have used self‐report methods of threat appraisal, including visual analogue scales (Symes & Putwain, 2016) and items from the revised Teachers Use of Fear Appeals Questionnaire (Putwain et al., 2019; Putwain & Symes, 2014) to explore challenge and threat states. Their findings demonstrated the mediating role of threat appraisal in determining students' response to fear appeals, with increased engagement and performance related to challenge, and less engagement and lower achievement related to threat (Putwain et al., 2017, 2023). Although somewhat limited relative to the established non‐cognitive factors already discussed, the examples cited offer evidence of the potential utility of threat appraisal in educational contexts, where as Tomaka et al. (1993, p. 258) note, ‘two simple cognitive judgements’ regarding situational demands and personal resources to cope with those demands have been shown to predict subsequent behaviour and performance.

The prevalence of motivated performance situations in education and learning contexts has been noted (Blascovich, 2008). However, much of the work investigating threat appraisal, both in education and in other fields, involves participants completing well‐defined tasks in a pre‐specified and limited timeframes temporally close to when threat appraisal measures are taken, where the individual is active—rather than passive—in that there is the capacity to actively engage and perform in the task, such as an arithmetic test used by Tomaka et al. (1993). Though valid, these tasks may not confer the same level of authenticity in a higher education context as the task of completing a degree programme. Though less well‐defined and following a more protracted trajectory than tasks traditionally featured in threat appraisal studies, completing an academic degree remains—it is argued—an active task that satisfies the core characteristics of a motivated goal‐relevant performance situation described by Blascovich (2008). That is, undertaking a degree is potentially stressful in nature, involves engagement in a task that is personally goal‐relevant for the student, and requires instrumental (cognitive, affective, behavioural) actions evaluated by the student and others against standards. Meeting these standards is necessary for the student to continue and attain the self‐relevant goal of successfully completing their degree, with the potential, it is suggested, for such top‐down goal‐directed attentional control to be compromised by threat states in a similar way to that suggested by Vine et al. (2013, 2015) in the case of performance in other tasks. Referring to attention control theory (Eysenck et al., 2007), Vine et al. (2013, 2015) argue that attentional control may underlie some of the processes by which threat appraisal influences task performance. That is, threat states induce anxiety, disrupting the balance in attentional control so that ineffective bottom‐up stimulus‐driven attentional control is dominant over more effective goal‐directed top‐down attentional control. Though not stated explicitly by Vine et al. (2013, 2015), the implication is that the balance in attentional control is maintained when a challenge state prevails.

### Present study

Despite its potential, threat appraisal, particularly using self‐report measures, has been the subject of only limited investigation in applied higher educational contexts. Self‐report threat appraisal therefore forms the primary focus for the present study, which seeks to investigate factors explaining student achievement and success and how these factors can inform interventions aimed at improving retention, attainment, and academic progression.

As a recent development, examples of self‐report threat appraisal measures reported in the literature are limited. Malkoc et al. (2023) and Feldhammer‐Kahr et al. (2021) each developed or adapted multi‐item scales measuring challenge and threat appraisals based on Drach‐Zahavy and Erez's (2002) longer 12‐item challenge‐threat scale comprising separate primary and secondary threat challenge appraisals. Usually demonstrating good reliability, both scales use multiple items to reflect the same fundamental principles of cognitive appraisal theory and primary demand and secondary resource appraisals captured in the two‐item cognitive appraisal ratio developed by Tomaka et al. (1993). Importantly however, using only single items to assess each of the demand and resource components of threat appraisal affords the cognitive appraisal ratio the arguable advantage, in the context of the present study at least, of greater utility in educational practice. As Vine et al. (2015) suggests, it is an example of valid and ‘expedient self‐report measures that can be easily collected in applied environments’ (p. 474). It has also been shown to align closely with, and be predictive of, cardiovascular indices of threat appraisal, where patterns of cardiac output and peripheral resistance differentiate distinct challenge and threat states (Moore et al., 2013; Vine et al., 2013).

**FIGURE 1:**
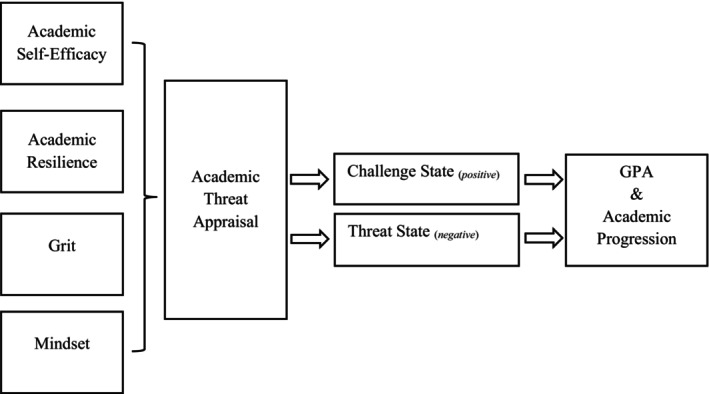
Hypothesized associations between antecedent factors (academic self‐efficacy, academic resilience, grit, mindset), academic threat appraisal, and outcome variables (academic progression and GPA).

Adapting the two‐item self‐report threat appraisal measure used by Vine et al. (2015), comprising items from Tomaka et al.'s (1993) cognitive appraisal ratio for use in an educational context (i.e., the Academic Threat Appraisal Ratio Scale, ATARS), the present study has two aims. First, with inconsistent and mixed findings in threat appraisal studies in educational contexts that rely on cardiovascular indices of threat appraisal, the study explores self‐report threat appraisal, reported to predict future performance (Vine et al., 2015), as a potentially scalable approach in a higher education to identify students at risk of dropping out through poor attainment and failure to progress. Findings have the potential to inform practice around supporting students and the design and implementation of interventions mitigating non‐continuation, improving attainment and retention. In line with findings from previous threat appraisal studies, significant differences in student attainment and academic progression according to self‐report primary and secondary appraisals of demands and resources involved in successfully completing their degree are anticipated, where a challenge state is associated with higher GPA and increased likelihood of academic progression to their second year at first attempt. Second, with limited extant research focusing on antecedents of threat appraisal in educational contexts (Smith et al., 2022), the study also aims to gain insight into the association between self‐report academic threat appraisal and relevant non‐cognitive factors and skills. Expanding our understanding in this way could lead to the development of educational practice that helps foster challenge, over threat, states. Examples of studies offeing evidence regarding antecedents of threat appraisal include Zandara et al. (2018) who report a negative association between dispositional optimism and threat state in a sample of university students, and Moore et al. (2014) who found that pre‐test manipulation of perceived required effort to complete a task significantly affected self‐report challenge‐threat states in a sample undergraduate students. Evidence of the association between threat appraisal and academic self‐efficacy (Putwain et al., 2015) and academic buoyancy—a construct similar to academic resilience—(Symes et al., 2015) has been reported in high school students. Given the emphasis on personal resources during secondary appraisal, significant positive associations between a challenge state and academic self‐efficacy, academic resilience, grit, and growth mindset are anticipated in the present study. Figure 1 represents the hypothesized associations explored in the study, where academic self‐efficacy, academic resilience, grit, and mindset serve as antecedents of threat appraisal, which in turn is associated with GPA and academic progression.

## METHOD

### Design

The study adopted a psychometric questionnaire‐based survey design employing self‐report measures of academic threat appraisal, academic self‐efficacy, academic resilience, grit, and mindset, together with student attainment and academic progression data. Correlational analysis was used to explore associations between self‐report academic threat appraisal and end of first year GPA (0%–100%), academic progression (pass/fail to progress to second year at first attempt), academic self‐efficacy, academic resilience, grit, and mindset, whilst a between‐subject approach was employed to examine differences in academic progression between student groups (i.e., challenge state vs. threat state). The potential of self‐report academic threat appraisal to predict GPA and academic progression was also examined as well as the potential of academic self‐efficacy, academic resilience, grit, and mindset to predict self‐report academic threat appraisal.

### Participants

Participants included 186 first‐year undergraduate students recruited from a university in the United Kingdom (*M*_age_ = 19.82, SD 3.65, non‐disclosed 5.9%; identifying as cisgender women 82.3%, identifying as cisgender men 16.7%, non‐disclosed 1%; White 68.3%, Asian or Asian British 16.7% [Indian 2.7%, Pakistani 8.6%, Bangladeshi 2.7%, Chinese 1.6%, any other Asian background 1%], Black or Black British 8% [Caribbean 1.6%, African 5.9%, any other Black background 0.5%], mixed‐race 3.2%, other ethnic group 1.6%, non‐disclosed 2.2%. All students were studying psychology single or joint with counselling or criminology honours degrees courses with comparable entry requirements (single honours 70.4%, joint honours 28.5%, not specified 1.1%). Participants had the option of not responding to any of the demographic questions. All non‐responses are reported as ‘non‐disclosed’ or ‘not specified’ and excluded as missing values from demographic data analysis. Similarly, if participants did not complete a construct measure, or progression or GPA data were not available, these were treated as missing values and excluded from that part of the analysis using the ‘exclude cases pairwise’ missing data function in SPSS. Missing data included academic threat appraisal 2.2%; academic resilience 1.6%; academic self‐efficacy 1.1%; grit 1.1%; mindset 1.1%; GPA 6.1%; and academic progression 9.6%. Power analysis performed to estimate sample size for a regression models with one and four predictors indicated a minimum sample of 81 and 125 respectively to achieve power of .8 with alpha of .05 and effect *f* ^2^ = 0.1 as per (Behnke & Kaczmarek, 2018) who report smaller effect sizes as typical in challenge threat index and performance studies.

### Study measures

As in other areas of psychological investigation (e.g., pain management, Campbell et al., 2013), studies of non‐cognitive factors and skills in education carry some risk of conceptual overlap. For instance, academic resilience, academic self‐efficacy, and grit all share perseverance as a common element in their conceptual understanding, whilst self‐efficacy has been described as a component in conceptualizations of resilience (Baluszek et al., 2023). The present study, it is argued, mitigates the risk of conceptual overlap by selecting constructs and associated construct measures that can be considered sufficiently distinct and are represented as such by the selected measures. Academic resilience, for example, includes reflection and emotional response components, whilst grit includes passion and goals in addition to perseverance.

#### The Academic Threat Appraisal Ratio Scale (ATARS)

Developed for the purposes of the present study, the ATARS (Appendix A) comprises two items adapted from the cognitive appraisal ratio (Tomaka et al., 1993) and the work of Vine et al. (2015): Item A (*Demands*), ‘On a scale of 1 to 6, how demanding do you think your degree course will be?’ (1 ‘Not at all’ to 6 ‘Extremely’ demanding); Item B (*Resources*), ‘On a scale of 1 to 6, how able are you to cope with the demands of your degree course?’ (1 ‘Not at all able’ to 6 ‘Extremely able’). Subtracting the score for Item A from the score for Item B produces a Demand Resource Evaluation Score (DRES), (Item_B_ – Item_A_ = DRES). The DRES represents academic threat appraisal on a scale −5 to 5. Positive scores, where perceived resources equal or outweigh perceived demands, is interpreted as a *challenge* state, and negative scores, where perceived demands outweigh perceived resources is interpreted as a *threat* state. Both DRES and academic challenge‐threat dichotomy (i.e., positive DRES = challenge state; negative DRES = threat state) were used as measures of academic threat appraisal in separate analyses conducted as part of the study.

#### The General Academic Self‐Efficacy (GASE) scale (Cassidy & Eachus, 2002)

The GASE scale is a 23‐item unidimensional context‐specific self‐report measure of academic self‐efficacy developed specifically for use in academic contexts with students. The measure includes negatively and positively worded items rated on a 9‐point scale from 1 ‘completely in disagreement’ to 9 ‘very much in agreement’. Example items include, ‘I know I have the ability to complete this course successfully’. Negatively worded items (e.g., ‘I have some doubts about my ability to grasp some of the topics taught on this course’) are reverse scored so higher total scores represent higher academic self‐efficacy. The GASE scale has a theoretical range of 23–207 with the original authors reporting good internal and external reliability (*α* = .86, *r* = .71) and construct validity for the scale (Cassidy & Eachus, 2002). Consistent with previous evidence, the present study reported high internal reliability for the GASE (*α* = .89).

#### The Academic Resilience Scale (ARS‐30) (Cassidy, 2016)

Using vignette methodology, the ARS‐30 is a 30‐item multidimensional context‐specific self‐report measure of academic resilience developed specifically for use in academic contexts with students. After considering a vignette depicting academic‐related adversity, items are rated on a 5‐point Likert scale from strongly agree to strongly disagree. Example items include, ‘I would see the situation as a challenge’. Scores are produced for total academic resilience (theoretical range 30–150) and for the three subscale dimensions of perseverance, reflecting and adaptive help‐seeking, and negative affect and emotional response. Negatively worded items (e.g., ‘I would just give up’) are reverse scored so higher scores represent greater academic resilience. The ARS‐30 has emerged in the recent literature as one of the most commonly used measures of academic resilience (Cui et al., 2023), with the original authors citing evidence supporting its reliability (total score *α* = .90, perseverance *α* = .83, reflecting and adaptive help‐seeking *α* = .78, and negative affect and emotional response *α* = .80) and construct validity (*r* = .49) (Cassidy, 2016). Consistent with previous evidence, the present study reported high internal reliability for the ARS‐30 (total score *α* = .90, perseverance *α* = .82, reflecting and adaptive help‐seeking *α* = .82, and negative affect and emotional response *α* = .80).

#### The 12‐Item Grit Scale (Duckworth et al., 2007)

The scale is a self‐report measure of grit comprising 12 items rated on a 5‐point scale from 1 ‘not at all like me’, to 5 ‘very much like me’. Example items include, ‘I have achieved a goal that took years of work’. Negative items (e.g., ‘I often set a goal but later choose to pursue a different one’) are reverse scored to higher scores represent more grit. Summing all items and dividing by 12 gives a total grit score, where 5 represents ‘extremely gritty’ and 1 represents ‘not at all gritty’. In a series of studies involving success outcomes (e.g., educational attainment, retention, and GPA) in samples including undergraduate students, the original authors cite convincing evidence for the reliability (*α* = .77 to .83) and validity (*r* = .14 to .68) of the Grit scale. Whilst the original authors identified two factors, consistency of interest and perseverance of efforts, they recommend using the total score, derived from the 12 items, based on its superior predictive validity. Consistent with previous evidence, the present study reported acceptable internal consistency reliability for the 12‐Item Grit scale (*α* = .79).

#### The Implicit Theories of Intelligence Scale (ITIS) (Dweck, 1999, 2006)

The ITIS is an 8‐item self‐report measure of fixed vs. growth mindsets reflecting an individual's implicit theory of intelligence as fixed or malleable/changeable. The measure includes four entity items (e.g., ‘You have a certain amount of intelligence, and you really can't do much to change it’) and four incremental items (e.g., ‘You can change even your basic intelligence level considerably’) focusing on intelligence. Items are rated on a 6‐point scale from 1'strongly agree’ to 6 ‘strongly disagree’, with Incremental items are reverse scored so that higher scores are indicative of a growth mindset. The ITIS yields good reliability (*α* = .82 to .97) and construct validity (Costa & Faria, 2018). Consistent with previous evidence, the present study reported high internal reliability for the ITIS (*α* = .92).

#### Attainment and academic progression

End of first‐year GPA (i.e., averaged mean mark across modules; range 0%–100%) provided the measure of student attainment, whilst academic progression at first attempt was measured according to whether students achieved a GPA of 40% or above after completing 120 course credits and therefore passed their first year and progressed to their second year without re‐assessment.^1^ Not progressing included students who had failed to achieve a GPA of 40% as a result of failing assessments or failing to submit work for assessment, or who had withdrawn from the course or interrupted their studies. Whilst previous studies have used self‐report GPA, some authors report concerns regarding the reliability of self‐reported GPA (Kuncel et al., 2005), prompting the use of *actual* GPA, retrieved from student data, in the present study to ensure greater reliability.

### Procedure

Following approval from the University of Salford Research, Innovation and Academic Engagement Ethics Approval Panel (application approval number HSCR15‐101), all participants received a participant information sheet and provided written informed consent before being invited to complete the battery of psychometric measures (paper‐and‐pencil method) during scheduled lectures and seminars. This included assurances regarding confidentiality of responses and the use of participants codes to anonymize data to help mitigate social desirability effects. All measures were completed in a single session, with an estimated time to complete of 15 min. Environmental conditions were consistent in that all participants completed the measures in the presence of other students and a tutor in similar teaching spaces and during normal teaching hours of 9 AM and 5 PM. The order in which measures were presented in the battery, and in theory completed by participants, was alternated across measures as a method of partial counterbalancing to help mitigate order effects. Whilst participants were not rewarded or compensated, measures were completed in allotted time during scheduled teaching sessions and participation in psychological research was relevant and of value to their studies. After completing the measures, participants received a debrief sheet including a reminder of the purpose of the study, details of data management, and contact details for academic support services and the principal investigator.

## RESULTS

Table 1 shows that more students (58.2%) reported a threat state than reported a challenge state (41.8%). Chi‐square and independent samples *t*‐tests confirmed a significant difference between the observed and expected proportions of students reporting challenge and threat states (*χ* ^2^ (1) = 4.945, *p* = .026) and that, as would be expected, students reporting a challenge state had significantly higher DRES than students reporting a threat state, with a large effect size (*t*(180) = 17.36, *p* < .001; *d* = 2.63).

**TABLE 1:** Academic threat appraisal summary statistics.

	*N*	%	Mean DRES (SD)	Min/max	Theoretical range
Challenge	76	41.8	0.37 (0.69)	0 to 4	0 to 5
Threat	106	58.2	−1.52 (0.75)	−4 to −1	−5 to −1
Total sample	182	100	−0.73 (1.18)	−4 to 4	−5 to 5

**FIGURE A1:**
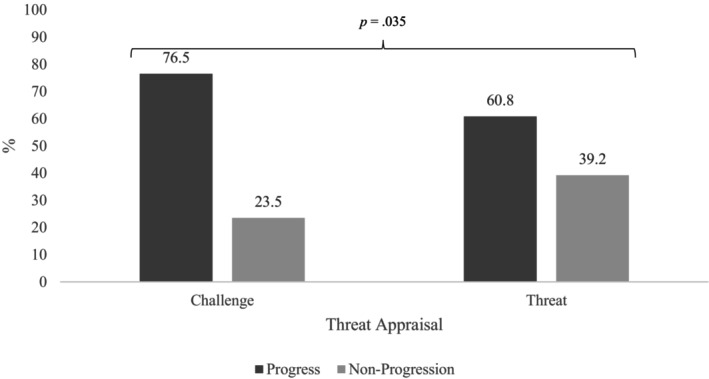
Progression at first attempt by academic challenge‐threat dichotomy.

**FIGURE A2:**
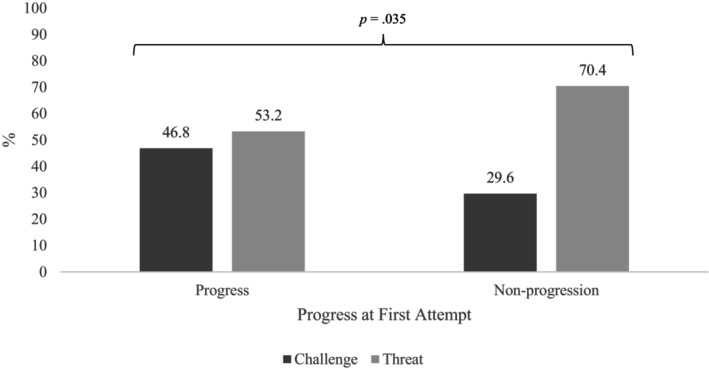
Academic challenge‐threat dichotomy by progression at first attempt.

Tables 2 and 3 (and Figures A1 and A2, Appendix B) illustrate a significant association between self‐reported academic threat appraisal and academic progression at first attempt. A challenge state was associated with a greater likelihood of students progressing at first attempt (76.5%), whilst a threat state was associated with a greater likelihood of students failing to progress at first attempt (70.4%) (*χ* ^2^ (1) = 4.445, *p* = .035).

**TABLE 2:** Academic progression at first attempt by academic challenge–threat dichotomy.

	Challenge		Threat	
	*N*	%	*N*	%
Progress	52	76.5	59	60.8
Non‐progression	16	23.5	38	39.2
Total	68	100	97	100

**TABLE 3:** Academic challenge‐threat dichotomy by progression at first attempt.

	Academic progression		Non‐progression	
	*N*	%	*N*	%
Challenge	52	46.8	16	29.6
Threat	59	53.2	38	70.4
Total	111	100	54	100

Correlation coefficients presented in Table 4 show small (Cohen, 1988) significant positive correlations between academic progression at first attempt and both DRES (*r*_pb_ = .18) and academic challenge–threat dichotomy (*r*_pb_ = .16). No significant correlations between academic threat appraisal indexes and GPA were found (*p* > .05).

**TABLE 4:** Correlation coefficients [95% CI] for academic threat appraisal with GPA and academic progression.

	GPA	Progress 1st attempt
1. DRES	.13 [−.03, .28]	.18** [.03, .33]
2. Challenge‐Threat Dichotomy	.06 [−.10, .22]	.16* [.01, .31]

*p < .05, **p < .01.

Correlation coefficients presented in Table 5 show significant small to medium (Cohen, 1988) positive correlations between DRES and academic self‐efficacy (*r* = .41), academic resilience (*r* = .29), grit (*r* = .16), and mindset (*r* = .16), and between academic challenge‐threat dichotomy and academic self‐efficacy (*r*_pb_ = .29), academic resilience (*r*_pb_ = .18), and mindset (*r*_pb_ = .16).

**TABLE 5:** Correlation coefficients [95% CI] for academic threat appraisal with non‐cognitive factors.

	Academic self‐efficacy	Academic resilience	Grit	Mindset
1. DRES	.41** [.28, .53]	.29** [.15, .42]	.16* [.01, .30]	.16* [.01, .29]
2. Challenge‐threat dichotomy	.29** [.15, .42]	.18** [.04, .32]	.12 [−.03, .26]	.16* [.02, .30]

*p < .05, **p < .01.

### Regression analysis

Based on the outcome of the correlational analysis (Tables 4 and 5) and the study's primary focus on academic threat appraisal, simple and multiple linear regression and non‐linear binary logistic regression analyses were conducted to determine (i) whether academic threat appraisal as a continuous DRES and as a dichotomous challenge‐threat categorical variable predicted student academic progression at first attempt, and (ii) whether academic self‐efficacy, academic resilience, grit, and mindset predicted academic threat appraisal measured as a continuous DRES and as a challenge‐threat dichotomy. Collinearity tests indicated that multicollinearity was not a concern, with all independent variables falling below even the most conservative VIF threshold of 3 and exceeding tolerance of 0.1 (academic self‐efficacy VIF = 2.42, Tolerance = .414; academic resilience VIF = 1.90, Tolerance = .527; grit VIF = 1.67, Tolerance = .60; mindset VIF = 1.21, Tolerance = .826).

- Results of the binary logistic regression were significant, indicating that academic threat appraisal as both a continuous DRES and as a challenge‐threat dichotomy were significant predictors of academic progression at first attempt. With DRES as the predictor, the model was statistically significant (*χ* ^2^ (1) = 5.637, *p* = .018), explaining 4.7% (Nagelkerke *R* ^2^ = .047, effect size = small, Cohen, 1988) of variance in academic progression at first attempt (*B* = .351, Wald = 5.305, *p* = .021, Exp(*B*) 1.421). With academic challenge‐threat dichotomy as the predictor, the model was also statistically significant (*χ* ^2^ (1) = 4.547, *p* = .033), explaining 3.8% (Nagelkerke *R* ^2^ = .038, effect size = small, Cohen, 1988) of variance in academic progression at first attempt (*B* = −.739, Wald = 4.366, *p* = .037, Exp(*B*) 2.093). Percentage classification accuracy predicting student academic progression at first attempt was 69.1% and 67.3% for DRES and academic challenge‐threat models respectively. Odds ratios for both models (DRES Exp(*B*) = 1.421 95% CI [1.054, 1.915]; academic challenge‐threat dichotomy Exp(*B*) = 2.093, 95% CI [1.047, 4.186]) indicated that the odds of students progressing at first attempt increased with increased challenge (over threat), such that higher DRES, reflecting a challenge state, was associated with increased likelihood of students progressing at first attempt, and students who reported a challenge [vs. threat] state were 2.093 times as likely to progress at first attempt.
- Multiple linear regression analysis used to predict DRES from academic self‐efficacy, academic resilience, grit, and mindset resulted in a significant model, explaining 18.8% of variance in DRES (*F*(4, 175) = 10.15, *R* ^2^ = .188, *p* < .001, effect size = medium, Cohen, 1988). However, only academic self‐efficacy was identified as a significant predictor of academic threat appraisal DRES (*B* = −.03, *t*(175) = 4.69, *p* < .001).
  Multiple binary logistic regre0ssion analysis predicting academic challenge‐threat dichotomy from academic self‐efficacy, academic resilience, grit, and mindset also resulted in a statistically significant model (*χ* ^2^ (4) = 18.578, *p* < .001), explaining 13.2% (Nagelkerke *R* ^2^ = .132, effect size = medium, Cohen, 1988) of variance in academic challenge‐threat dichotomy. Again, only academic self‐efficacy was identified as a significant predictor of academic challenge‐threat dichotomy (*B* = .036, Wald = 9.23, *p* = .002, Exp(B) 1.037). Percentage classification accuracy predicting academic challenge‐threat dichotomy was 65%. Odds ratio for the model (Exp(*B*) = 1.037, 95% CI [1.013, 1.061] indicated that the odds of students reporting a challenge state increased with increased academic self‐efficacy.

## DISCUSSION

The study was prompted by a need for practical and scalable approaches addressing issues relevant to improving student retention in higher education and the potential—with only relatively limited extant evidence in educational contexts (Smith et al., 2022)—of self‐report threat appraisal to offer unique insight into student attainment and academic progression. The two principal aims of the study were to first establish whether self‐report academic threat appraisal was associated with, and a predictor of, student attainment and academic progression, and second, given the relative paucity of research examining antecedents of threat appraisal (Moore et al., 2014) and specifically in higher education, to investigate the relationship between self‐report academic threat appraisal and non‐cognitive factors commonly explored in the context of student performance.

With regards to the first aim, findings revealed a significant association between self‐report academic threat appraisal and student attainment and academic progression. A significantly greater proportion of students eliciting a challenge state at the start of their degree course progressed to their second year at first attempt compared with students who had elicited a threat state. Furthermore, of those students who failed to progress to their second year at first attempt, a significantly greater proportion had elicited a threat state at the start of their degree. In addition, academic threat appraisal, measured as both a DRES and as a challenge‐threat dichotomy, was identified as a significant predictor of academic progression at first attempt, where a challenge state predicted a greater likelihood of academic progression to the second year at first attempt. The variance in academic progression accounted for by self‐report threat appraisal (3.8% and 4.7%) was comparable to the level of explained variance in academic performance (3.9%) using physiological measures of threat appraisal reported by Seery et al. (2010). Percentage accuracy classification models for both DRES and academic challenge‐threat dichotomy exceeded the suggested minimum benchmark of 67% reported in other fields of psychology to be ‘clinically useful’ predictive accuracy (Forsell et al., 2022). Whilst not definitive, this benchmark suggests that findings reported here may have practical significance in informing decisions in educational practice. The association between a challenge state and improved performance in a motivated performance situation is relatively well evidenced in other fields and contexts (e.g., Moore et al., 2013; Vine et al., 2013, 2015), and to some extent in education (e.g., Malkoc et al., 2023; Putwain et al., 2017, 2023; Seery et al., 2010). This association has not however, to the author's knowledge, previously been demonstrated in a similar higher education context using the two‐item cognitive appraisal ratio (Tomaka et al., 1993) adapted to garner self‐report academic threat appraisal in university students, and utilizing objectively recorded GPA rather than self‐report GPA (shown previously to be unreliable, Kuncel et al., 2005). Whilst no significant association between DRES or academic challenge‐threat dichotomy and end of first‐year GPA was found, a similar lack of association has been reported elsewhere using cardiovascular indices of threat appraisal and measures of academic performance (e.g., Smith et al., 2022). Smith et al. (2022) suggests a number of factors that might explain such ‘indifferent findings’ or the lack of association, including the variety of tasks used in studies, students' relative interest in the task, delay between threat appraisal evaluation and performance task, sensitivity of measures, and the potential that threat appraisal itself facilitates performance through compensatory strategies and increased effort. It is possible that one or more of these factors contributed in part to the lack of association reported in the present study, but it is also suggested that GPA alone fails to capture fully all aspects involved in determining student academic progression. On this point, Smith et al. (2022) consider the possible inadequacy of performance as the singular predicted outcome, highlighting the potential value of exploring other relevant outcome factors that might reveal important interactions with threat appraisal and performance. Malkoc et al. (2023) have previously reported an indirect effect of self‐report threat appraisal on performance, with unpleasant emotions as the mediating factor. Motivation, underpinned by expectancy value theory which posits that increased motivation is a function of both valuing the task and an expectation of successful completion of the task (Wigfield & Eccles, 2000), is another plausible candidate for a mediating factor in the relationship between threat appraisal and academic performance.

With regards to the study's second principal aim of exploring non‐cognitive antecedents of self‐report academic threat appraisal, significant positive correlations were reported between a challenge state and academic self‐efficacy, academic resilience, grit, and mindset. However, only academic self‐efficacy was identified as a significant predictor of academic threat appraisal, where greater academic self‐efficacy predicted a challenge state. Despite Smith et al. (2022) previously reporting a lack of significant association between cardiovascular indices of threat appraisal and academic self‐efficacy, a significant association has been reported in other studies using self‐report methods (Putwain et al., 2015), so was anticipated. The relationship can, it is suggested, be explained in terms of the reliance of demand resource evaluations (i.e., threat appraisal) on secondary appraisals of perceived personal resources, which would certainly include evaluations of personal capabilities and competence—defined within the self‐efficacy construct in particular (Gallagher, 2012)—as key determinants of successful outcomes. Furthermore, in existing evidence demonstrating the relevance of non‐cognitive factors to student performance (Frantz et al., 2022; Khine, 2016), self‐efficacy emerges as the strongest non‐cognitive predictor of GPA (Robbins et al., 2004). The lack of predictive power of academic resilience, grit, and mindset reported in the present study may therefore be explained by evidence of a dominant influence of academic self‐efficacy in the regression models and should not lead to their dismissal as relevant factors in understanding threat appraisal in students. In fact, these constructs, reflecting adaptive responses in adverse academic situations, perseverance and passion, and beliefs regarding ability as fixed or something that can be further developed, are likely to add significant insight into attempts to model academic threat appraisal, warranting further investigation. Future studies should integrate mediation and moderation approaches into their design, considering both direct and indirect effects of these non‐cognitive factors on threat appraisal, employ longitudinal designs with multiple data points capturing the temporal trajectory of academic threat appraisal, and include other pertinent student success outcome variables beyond GPA and academic progression.

The study has some limitations worthy of highlighting and considering when both interpreting findings and planning future investigations. Students classified as not progressing at first attempt did not constitute a homogenous group. The group included students who had failed assessments, failed to submit assessments, withdrawn permanently from the course at different stages, or who had interrupted their studies. It is plausible that academic threat appraisal interacts with the reason for non‐progression where, for instance, its association with performance means that it explains student failure due to poor performance but does not explain failure to submit assessments or student withdrawal, where other factors such as intention to drop out could be more prevalent. Finally, students with missing academic progression data (9.6%) were excluded from analysis involving progression. Whilst doing so was considered justified, it is probable that these students had withdrawn from their course and could, arguably, have been included in the non‐progression group. Future studies should therefore consider a finer‐grained analysis of how academic threat appraisal relates to and interacts with the range of circumstances governing student non‐progression.

There are two suggested avenues for future academic threat appraisal studies to focus their efforts. These involve the need for further validation of the ATARS as a measure of threat appraisal in higher education and evidence regarding optimal timing for students to complete the measure. For instance, validation studies should subject the ATARS to review by content experts (Jenn, 2006), in this case, university students, to ensure that students understand the question items as intended, and that this understanding is consistent across students and student groups, including cultural variations. Furthermore, studies incorporating traditional physiological measures of threat appraisal and existing multi‐item self‐report threat appraisal measures, such as those developed by Malkoc et al. (2023) and Feldhammer‐Kahr et al. (2021), alongside the ATARS will offer further insight into the validity of the ATARS as a newly developed self‐report measure of academic threat appraisal in educational contexts. Whilst there is no question that the ATARS should be completed prospectively in anticipation of completing the degree course (i.e., the motivated performance situation), test‐retest reliability studies would help determine if there is a critical point at which students should complete the measure when, for example, there is adequate understanding of the task. Finally, previously demonstrated in other fields (Moore et al., 2013), exploring the potential for manipulating academic threat appraisal in higher education to foster a greater propensity for challenge over threat is another valuable avenue for future studies in the area.

## CONCLUSION

In line with studies in other fields (e.g., More et al., 2013; Vine et al., 2015) and in different educational contexts and levels (e.g., Putwain et al., 2023), the present study provides evidence supporting self‐report academic threat appraisal as a significant factor in student attainment and academic progression in higher education. Findings suggest that self‐report academic threat appraisal, measured using the ATARS, may offer a relatively simple, valid, and scalable solution for early screening of students, enabling targeted student support. Thus, educators and future researchers should consider recruiting all new students to complete the ATARS, the outcome of which might then form the basis to reflection, education, intervention, support, and practice and policy revision involving both students and staff. Significant associations with academic threat appraisal suggest academic resilience, grit, mindset, and in particular academic self‐efficacy, as possible underlying mechanisms explaining the influence of threat appraisal on attainment and academic progression. For this reason, support focused on developing students' awareness, understanding, and skills in these areas, as well as direct manipulation of threat appraisal previously demonstrated by Moore et al. (2013), may help foster threat appraisals more likely to lead to challenge—rather than threat— states, helping improve attainment, academic progression, and ultimately, retention in higher education. The ATARS may offer a suitable tool by which to evaluate such interventions geared towards promoting completing a degree as an achievable challenge, rather than a threat to be avoided. This could include adapting the ATARS for use with incremental tasks such as completing a dissertation, an exam, group project, or presentation. Importantly, the responsibility for embedding and engaging with a ‘challenge culture’ should be shared between those likely to benefit, which includes staff, students, and senior leadership as key stakeholders. Whilst not notwithstanding the value of other established non‐cognitive factors and skills, screening students using the ATARS as a measure of self‐report academic threat appraisal could prove to be a small hack with big benefits for higher education.

## AUTHOR CONTRIBUTIONS

**Simon Cassidy:** Conceptualization; investigation; writing – original draft; methodology; writing – review and editing; formal analysis.

## CONFLICT OF INTEREST STATEMENT

The authors declare no conflict of interest.

## Data Availability

The data that support the findings of this study are available on request from the corresponding author. The data are not publicly available due to privacy or ethical restrictions.

## Footnotes

^1^
Academic progression is determined using UK GPA pass/fail standards (i.e., 40% and 120 credits completed).

## APPENDIX A Academic Threat Appraisal Ratio Scale (ATARS)

Thinking about your degree course, answer the following two questions by placing a tick in one of the boxes from 1 (‘Not at all’) to 6 (‘Extremely’):

**Table:**

A. On a scale of 1 to 6, how demanding do you think your degree course will be?	Not at all demanding	1	2	3	4	5	6	Extremely demanding
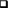
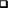
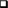
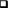
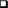
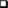
B. On a scale of 1 to 6, how able are you to cope with the demands of your degree course?	Not at all able	1	2	3	4	5	6	Extremely able
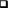
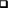
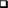
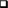
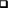
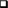

*Scoring*: Subtracting the score for item A (demands) from the score for item B (resources) gives a demand resource evaluation score (DRES), i.e., Item_B_ – Item_A_ = DRES.

A positive score (where resources equal or outweight demands) is interpreted as an academic *challenge* state, and a negative score (where demands outweigh resources) is interpreted as an academic *threat* state.

Note: It is suggested that the ATARS can be adapted by, for instance, adding ‘this year’, ‘this semester’, etc. to the end of each question to specify a particular upcoming part of the academic course, or substituting ‘degree course’ for ‘exam’, dissertation’, ‘presentation’, etc. to specify a particular academic task or situation.
